# Validity, reliability, measurement invariance, and equipercentile linking of the depression anxiety stress scale-21 in the youth population in Singapore

**DOI:** 10.3389/fpsyg.2025.1563190

**Published:** 2025-06-19

**Authors:** Edimansyah Abdin, Bernard Tan, Sherilyn Chang, Ellaisha Samari, Brian Tan, Charmaine Tang, Janhavi Ajit Vaingankar, Swapna Kamal Verma, Mythily Subramaniam

**Affiliations:** ^1^Institute of Mental Health, Singapore, Singapore; ^2^Department of Psychosis, Institute of Mental Health, Singapore, Singapore

**Keywords:** depression anxiety stress scale, DASS-21, validity, reliability, equipercentile linking

## Abstract

**Objective:**

The current study aims to examine the structural and convergent validity, reliability, and measurement invariance of the Depression Anxiety Stress Scales Short Form (DASS-21) across age and gender among the youths in Singapore. Additionally, it aims to provide a simple and reliable method for converting the DASS-21 Depression and DASS-21 Anxiety to the Patient Health Questionnaire 8-item (PHQ-8) and Generalized Anxiety Disorder 7-item (GAD-7) scores using an equipercentile linking method.

**Methods:**

A total of 2,600 respondents were recruited from a National Youth Mental Health Study.

**Results:**

The confirmatory factor analysis (CFA) confirmed that the original three-factor model fits our data. Cronbach’s alpha coefficients for the depression, anxiety, and stress subscales were 0.91, 0.87, and 0.89, respectively. Multiple CFA across age and gender showed that the configural, metric, and scalar measurement invariance models strongly support the three-factor model. The intraclass correlation coefficient (ICC) between the raw and converted PHQ-8 and GAD-7 scores support that the DASS-21 subscale scores are practically exchangeable with the PHQ-8 and GAD-7.

**Conclusion:**

These findings suggest that the DASS-21 is a valid tool for measuring depression, anxiety, and stress among the youths in Singapore.

## Introduction

Since the COVID-19 pandemic, mental health issues among children and youths have drawn considerable concern and attention among global researchers (Hossain et al., 2022). A national survey of children aged 6–17 years that was conducted in the US from 2003 to 2012 reported that the incidence of depression or anxiety increased from 5.4% in 2003 to 8.4% in 2011 to 2012 (Bitsko et al., 2018). Meanwhile, a meta-analysis of 29 studies involving more than 80 thousand youth globally has suggested that the prevalence of depression and anxiety symptoms doubled during the COVID-19 pandemic as compared to the pre-pandemic period (Racine et al., 2021). Several studies have suggested the COVID-19 pandemic had a negative impact on the psychological wellbeing of children and youths due to lockdown restrictions, school closures, increased family stress, and decreased peer interactions, all potential precipitants of psychological distress and mental health difficulties in youth (Sergi et al., 2023; Brooks et al., 2020; Racine et al., 2021). As the number of children and adolescents with depression and anxiety globally is expected to increase after the COVID-19 pandemic, it warrants early detection of their mental health problems with a reliable and valid screening tool that could lead to timely intervention and prevention in those affected by these conditions. One of the most widely used instruments is the Depression Anxiety Stress Scale 21 (DASS-21) (Lovibond and Lovibond, 1995). The DASS-21 is a screening instrument originally developed by Lovibond and Lovibond (1995), which has 42 items. It is used to distinguish between depression, anxiety, and stress as distinct states of negative emotion. The DASS-21 is a shortened version of the full version of the DASS-42. The instrument consists of three subscales: depression, anxiety, and stress, where each subscale is measured by seven items. A recent systematic review and meta-analysis in the youth population (Dwight et al., 2024) has shown that the DASS-21 has good internal consistency and appears to have a strong convergent validity with other scales, including Beck Depression Inventory (BDI) and Generalized Anxiety Disorder 7 item (GAD-7). The Cronbach alpha obtained by previous studies was high and ranged from 0.72 to 0.93 for the depression subscale, 0.75 to 0.90 for the anxiety subscale, and 0.70–0.94 for the stress subscale (Dwight et al., 2024; Anghel, 2020; Jovanović et al., 2021; Le et al., 2017; Mellor et al., 2015; Norton, 2007; Silva et al., 2016; Tully et al., 2009). Furthermore, the structural validity has been reported among children and adolescents to be adequately valid for a three-factor (Lovibond and Lovibond, 1995; Chen et al., 2024), bifactor (Moore et al., 2017), two-factor (Silva et al., 2016; Yap and Lee, 2023), and one-factor structure (Shaw et al., 2017). However, the factor structure of the DASS-21 has yielded inconsistent findings across international studies. The original factor structure of the DASS-21 by Lovibond and Lovibond (1995) has been supported among adolescents in four countries, including in Australia, Chile, China, and Malaysia (Mellor et al., 2015), and among undergraduate students in the United States across four racial groups, including African-American, Asian, Caucasian, and Hispanic/Latino (Norton, 2007). Other studies have supported a one factor structure among children and adolescents in Australia (Shaw et al., 2017; Patrick et al., 2010). Hence, given that numerous studies across different countries support varying factor structure, it is imporntant to validate the factor structure of this instrument within our local sample.

Singapore is an island city-state in Southeast Asia with a multi-ethnic Asian population of about 6 million people in 2024. The population comprises Chinese (74.3%), Malays (13.4%), Indians (9.1%), and other ethnic groups (3.2%). Previous studies in Singapore have highlighted a higher prevalence of mental disorders in the youth population (18–34 years) (Chong et al., 2012; Subramaniam et al., 2019). In order to detect and monitor the levels of depression, anxiety and stress in the youth population efficiently, it is important to have a valid and reliable screening instrument. To date, however, studies examining the validity and reliability of the DASS-21 among the Asian youth population, are currently lacking. There is also limited evidence about the measurement invariance of the DASS-21 instrument across subgroups in the Singapore context, especially across age and gender and whether the scores are exchangeable with common measures of depression and anxiety such as the Patient Health Questionnaire 8 item (PHQ-8) and GAD-7 scales. The current study aimed to examine the validity, reliability, and measurement invariance of the DASS-21 across age and gender in the youth population in Singapore. We also aimed to provide a simple and reliable linking method for converting the DASS-21 Depression and DASS-21 Anxiety to the PHQ-8 and GAD-7 scores, respectively using a conversion table derived from equipercentile linking method.

## Methods

### Study design

The present study is part of the National Youth Mental Health Study, which was a cross-sectional epidemiological study conducted between October 2022 and June 2023 among youths aged 15 to 35 years living in Singapore (Subramaniam et al., 2025). Respondents were included in the study if they were a Singapore Citizen or Permanent Resident (PR), aged 15 to 35 years, literate in English, Mandarin, Bahasa Melayu, or Tamil, and able to provide written informed consent (and consent from a legally acceptable representative for those below age 21). Those who were unable to complete the assessment on their own and were unable to provide written consent were excluded from the study. The study was approved by the institutional ethics review boards of participating institutions [National Healthcare Group Domain Specific Review Board (DSRB) (NHG DSRB Reference Number: 2021/00562)]. All respondents provided written informed consent and in the case of participants who were less than 21 years of age, a written informed consent was taken from their parent/legally acceptable representative.

### Questionnaires

The Depression Anxiety and Stress Scales 21 (DASS-21) (Lovibond and Lovibond, 1995) contains 21 items covering three symptoms of depression, anxiety, and stress. The scale assesses the symptoms in the past week and uses a 4-point Likert scale (from “did not apply to me” to “applied to me very much”). The DASS-21 subscale scores can be calculated by summing the scores of the items of each subscale and multiplying them by 2. The subscales scores can range from 0 to 42 (Lovibond and Lovibond, 1995).

The WHODAS 2.0 questionnaire contains 12 items that assess disability in various domains of functioning including cognition, mobility, self-care, getting along, life activities, and participation during the preceding 30 days. Each item uses a 5-point Likert-type scale to reflect the level of difficulty, starting with “no difficulty” and increasing in an ordered fashion from “mild,” “moderate,” “severe” to “extreme or cannot do.” A simple scoring can be generated by assigning each item a score ranging from 0 (mild) to 4 (extreme or cannot do) –which are then summed up with total scores ranging from 0 to 48.

The PHQ-8 is a self-administered scale to measure depressive symptoms in the past 2 weeks using a 4-point Likert-type scale from 0 = not at all to 3 = nearly every day. Total scores range from 0 to 24, where scores of 10 and above indicate current depression (Kroenke et al., 2009). The GAD-7 was used to measure anxiety symptom severity in the past 2 weeks using a 4-point Likert-type scale. The total scores can range from 0 to 21 (Spitzer et al., 2006).

Socio-demographic information including age, gender, and ethnicity were also collected from the participants.

### Statistical analysis

All analyses were performed in RStudio software version 2022.07.2. We adopted the standard approach for assessing the psychometric properties of the DASS-21 by assessing the internal consistency, structural validity, measurement invariance and convergent validity. Internal consistency was examined using Cronbach alpha coefficient (Cronbach, 1951). Cronbach alpha ≥0.7 is usually regarded as acceptable (Cronbach, 1951). Prior to CFA, multivariate normality was tested using Mardia’s test, and a clear violation of the multivariate normal distribution assumption for each scale was found (*p* < 0.001). Hence, the structural validity of the scale in terms of factor structure models proposed by previous studies was examined through confirmatory factor analysis (CFA) using diagonal weighted least squares estimator. The following models were tested, including the one-factor model (Model 1) (Shaw et al., 2017), a two-factor model (Model 2) (Silva et al., 2016; Yap and Lee, 2023), a three factor model (Model 3) (Lovibond and Lovibond, 1995; Chen et al., 2024), and a bifactor model (Model 4) (Moore et al., 2017). Subsequently, Omega coefficient was calculated for each scale using the best factor structure model found in the CFA.

The goodness of fit indices of each model was assessed using the three indices, including root mean square error of approximation (RMSEA), comparative fit index (CFI), and Tucker-Lewis index (TLI). The CFI values above 0.95 and TLI values above 0.90 are considered to be of excellent fit, while RMSEA values below 0.8 are considered to be acceptable (Browne and Cudek, 1993). The overall model fit was considered an adequate fit if at least two of these three indices met their respective cut-off point (Ferro and Boyle, 2013; Ferro and Speechley, 2013; Tompke et al., 2018). Measurement invariance of the DASS-21 across age groups (15–24 years vs. 25–33 years) and gender were tested through multiple-group CFA (MGCFA). The MGCFA began by performing CFA independently in each subgroup to establish the appropriateness of a baseline model. Subsequently, the MGCFA was conducted to establish full measurement invariance across subgroups. Three measurement invariance models were tested including (1) the configural model (i.e., to examine whether the factor structure of the DASS-21 is similar across subgroups); (2) the metric model or weak invariance (i.e., to examine whether the factor loadings are similar across subgroups); and (3) the scalar model or strong invariance (i.e., to examine whether the factor structure, loadings and intercepts are similar across subgroups) (Fischer and Karl, 2019). Each measurement invariance model was considered acceptable if two or more changes in the following criteria indices were satisfied: ΔCFI ≤0.01, ΔTLI ≤0.01, or ΔRMSEA ≤ 0.015 (Kimber et al., 2015; Chen, 2007; Cheung and Rensvold, 2002). The convergence validity between the DASS-21 subscales, WHODAS 2.0, PHQ-8 and the GAD-7 was examined using Pearson’s (r) correlation coefficients. We used the following categories for evidence of convergent validity: >0.6, very strong; ≥0.5 to <0.6, strong; <0.5 to ≥ 0.3, moderate; and <0.3, weak (Papaioannou et al., 2011). To provide a cross-walk score from DASS Depression to PHQ-8 and DASS Anxiety to GAD-7 scores, an equipercentile linking method with log-linear smoothing was used (Kolen and Brennan, 2014). This method needs both scales to measure the same construct and to have at least a moderate correlation (r = 0.3) (Dorans, 1990). The Structural Equation Modelling (SEM)-based linking method was also examined to account for measurement errors of the scale (DiStefano et al., 2009). In this method, factor scores were generated with the lavPredict function and then cross walked using a linear linking method. We evaluated the agreement between the raw and converted scores using the intraclass correlation coefficient (ICC) and the Bland–Altman plot. The ICC values were interpreted as poor (ICC < 0.40), fair (ICC = 0.40–0.59), good (ICC = 0.60–0.74), or excellent (ICC = 0.75–1.0) (Hallgren, 2012). The limits of agreement (LOA) at 1.96 standard deviations from the mean difference were used in the Bland–Altman plot to describe the agreement between the raw and converted scores (Bland and Altman, 1986).

## Results

### Socio-demographic characteristics

Sample characteristics are presented in Table 1. A total of 2,600 respondents completed the study. Most of the respondents (97.9%) completed the English version, followed by Chinese (2%) and Malay (0.1%) versions of the DASS-21. The sample comprised 50.2% female and 49.8% male respondents. The mean age of the overall sample was 25.7 years (SD = 6.0; range = 15–35 years), 70.9% were Chinese, 16.6% were Malays, 9.3% were Indians, and 3.1% belonged to other ethnicities. A total of 2,567 respondents who completed English version of the DASS-21 were included for the study.

**Table 1:** Sociodemographic characteristics of the sample (*n* = 2,600).

Variables	*N* (sample)	Weighted %
Age (mean, SD)	25.7	6.0
15–19	632	18.8
20–24	672	21.2
25–29	634	25.4
30–35	662	34.6
Gender		
Male	1,381	49.8
Female	1,219	50.2
Ethnicity		
Chinese	1,313	70.9
Malay	658	16.6
Indian	506	9.3
Others	123	3.1

### Structural validity

**Figure 1:**
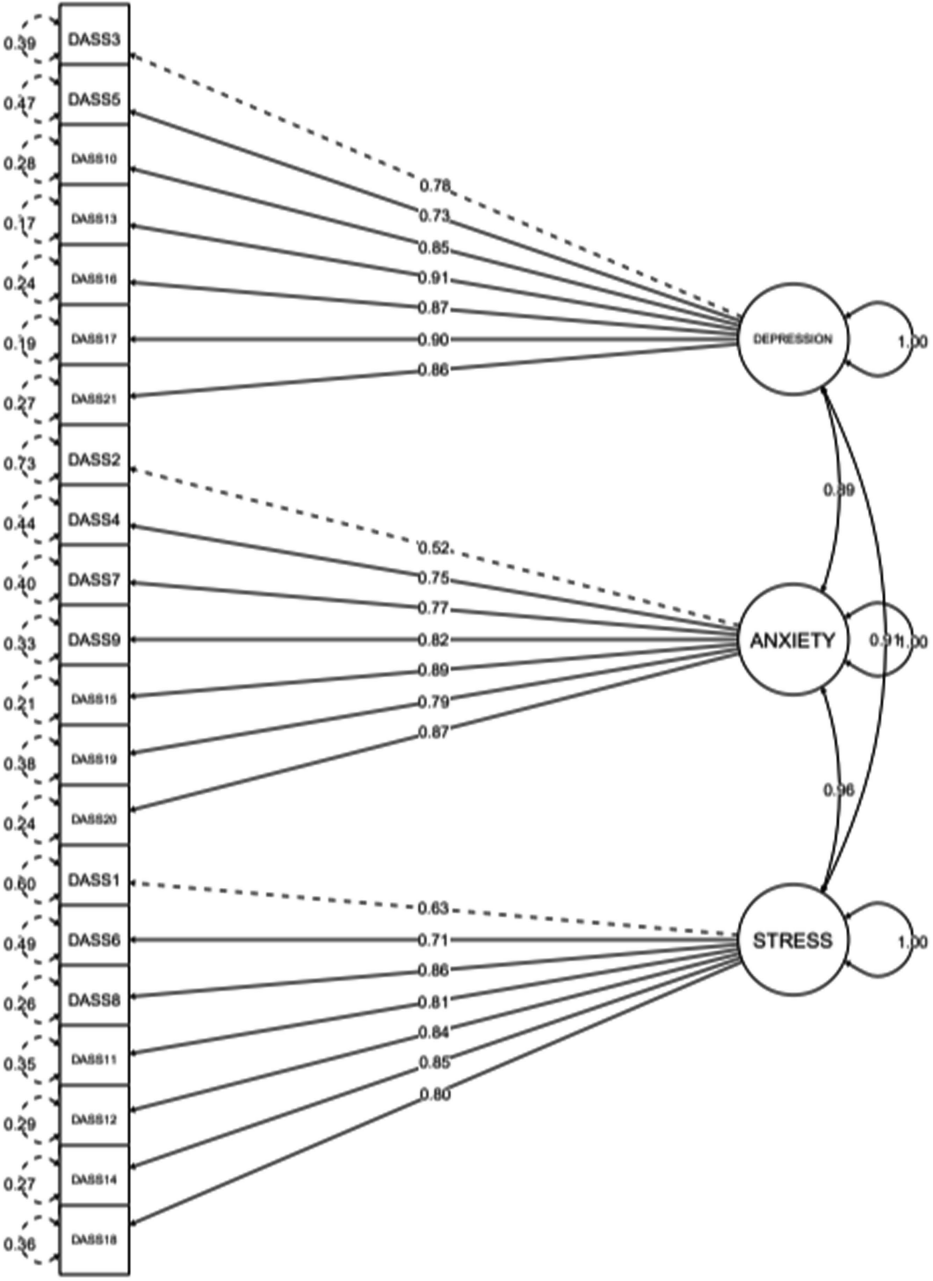
Factor structure of the DASS-21.

Four hypothetical models were tested using CFA to examine the factor structure of the DASS-21 are shown in Table 2. The goodness-of-fit indices of the original 3-factor model (Model 3) (*χ*^2^ = 2367.969, df = 186, CFI = 0.9938, TLI = 0.9929, RMSEA = 0.0676) were a better fit than Model 1 and Model 2. The factor loadings in Model 1 were all significant (*p* < 0.001) and ranged from 0.73 to 91 for depression, 0.52 to 0.89 for anxiety, and 0.63 to 0.86 for stress. We also observed that the goodness-of-fit indices for a bifactor model (Model 4) were a slightly better fit than that of Model 3 (*χ*^2^ = 111835.76, df = 168, CFI = 0.995, TLI = 0.994, RMSEA = 0.062). However, we found that Model 4 failed to converge due to negative error variances. Hence, Model 3 was chosen as the best-fitting model and used further for measurement invariance testing (Figure 1). The Omega coefficient for depression, anxiety, and stress was 0.92, 0.88, and 0.89, respectively.

**Table 2:** Confirmatory factor analysis results of factor structure of the DASS-21.

	Chi Square	df	X/df	CFI	TLI	RMSEA
Model 1	3106.468	189	16.4363	0.9916	0.9907	0.0776
Model 2	2430.992	188	12.9308	0.9936	0.9928	0.0682
Model 3	2367.969	186	12.7310	0.9938	0.9929	0.0676
Model 4	1835.762	168	10.9272	0.9952	0.9940	0.0622

### Measurement invariance across age groups and gender

#### Age

Results of measurement invariance tests of the DASS-21 using MGCFA across age and gender are shown in Table 3. The factor structure of the DASS-21 had an adequate fit to the data for different age groups (15–24 years: RMSEA = 0.061, CFI = 0.995, TLI = 0.994 and 25–33 years: RMSEA = 0. 077, CFI = 0.992, TLI = 0.991). In the configural model, the model demonstrated adequate fit across age groups (RMSEA = 0.069, CFI = 0.993, TLI = 0.994). We then further analysed the measurement invariance in terms of its metric and scalar models. We found that the changes in the three fit indices between the configural and metric models (ΔCFI = 0.001, ΔTLI = <0.001, ΔRMSEA = 0.001) and between the metric and scalar models (ΔCFI <0.001, ΔTLI = −0.001, ΔRMSEA = 0.003) suggest that the fit of the metric and scalar models was satisfactory. We also found that the fit indices for the configural, metric, and scalar models were satisfactory when testing measurement invariance between individuals aged 15–18 years and those aged 19–33 years (ΔCFI ≤0.01, ΔTLI ≤0.01, or ΔRMSEA ≤ 0.015). Hence, this suggests that the configural, metric and scalar measurement invariance models across different age groups were supported in this study.

**Table 3:** The goodness of fit indices of measurement invariance models.

	Ch-Squared	df	CFI	∆ CFI	TLI	∆TLI	RMSEA	∆RMSEA
1. Age								
15–24	1193.711	186	0.995		0.994		0.061	
25–33	1425.39	186	0.992		0.991		0.077	
Configural	2619.165	372	0.994		0.993		0.069	
Loadings	2699.257	390	0.993	0.001	0.993	0.000	0.068	0.001
Intercepts	2725.519	429	0.993	0.000	0.994	−0.001	0.065	0.003
2. Age								
15–18	1981.633	186	0.994		0.993		0.069	
19–33	512.461	186	0.995		0.994		0.057	
Configural	2493.884	372	0.994		0.993		0.067	
Loadings	2632.207	390	0.994	0.000	0.993	0.000	0.067	0.000
Intercepts	2584.715	429	0.994	0.000	0.994	−0.001	0.063	0.004
3. Gender								
Male	1246.856	186	0.995		0.994		0.065	
Female	1239.62	186	0.994		0.993		0.069	
Configural	2486.484	372	0.994		0.993		0.067	
Loadings	2588.558	390	0.994	0.000	0.993	0.000	0.066	0.001
Intercepts	2580.999	429	0.994	0.000	0.994	−0.001	0.063	0.003

#### Gender

For gender, the factor structure of the DASS-21 had adequate fit to the data within male and female subgroups (male: RMSEA = 0.065, CFI = 0.995, TLI = 0.994 and female: RMSEA = 0. 069, CFI = 0.994, TLI = 0.993). In the configural model, the model demonstrated adequate fit across gender (RMSEA = 0.067, CFI = 0.994, TLI = 0.993). The model was then tested for metric and scalar models. We found that the changes in the three fit indices between the configural and metric models (ΔCFI <0.001, ΔTLI = <0.001, ΔRMSEA = 0.001) and between the metric and scalar models (ΔCFI <0.001, ΔTLI = −0.001, ΔRMSEA = 0.003) suggests that the fit of the metric and scalar models was satisfactory. Hence, this suggests that the configural, metric and scalar measurement invariance models across gender were also supported in this study.

#### Reliability

Internal reliability showed that the DASS-21 subscales have good internal consistency and reliability in the current sample. The Cronbach’s alpha coefficients were acceptable for depression (*α* = 0.91), anxiety (*α* = 0.87), and stress subscales (*α* = 0.89). The Omega reliability index has also supported these findings.

#### Convergent validity

The correlation coefficient between the WHODAS 2.0 total scores with the DASS-21 subscales was moderate (depression, *r* = 0.46, anxiety, *r* = 0.46, and stress, *r* = 0.43). Among the subset of participants with completed PHQ-8 and GAD-7 (*n* = 889), the correlation coefficient between PHQ-8 and DASS-21 depression subscale scores was 0.63, while the correlation between GAD-7 and DASS-21 anxiety subscale scores was 0.59.

#### Equipercentile linking

**Figure 2:**
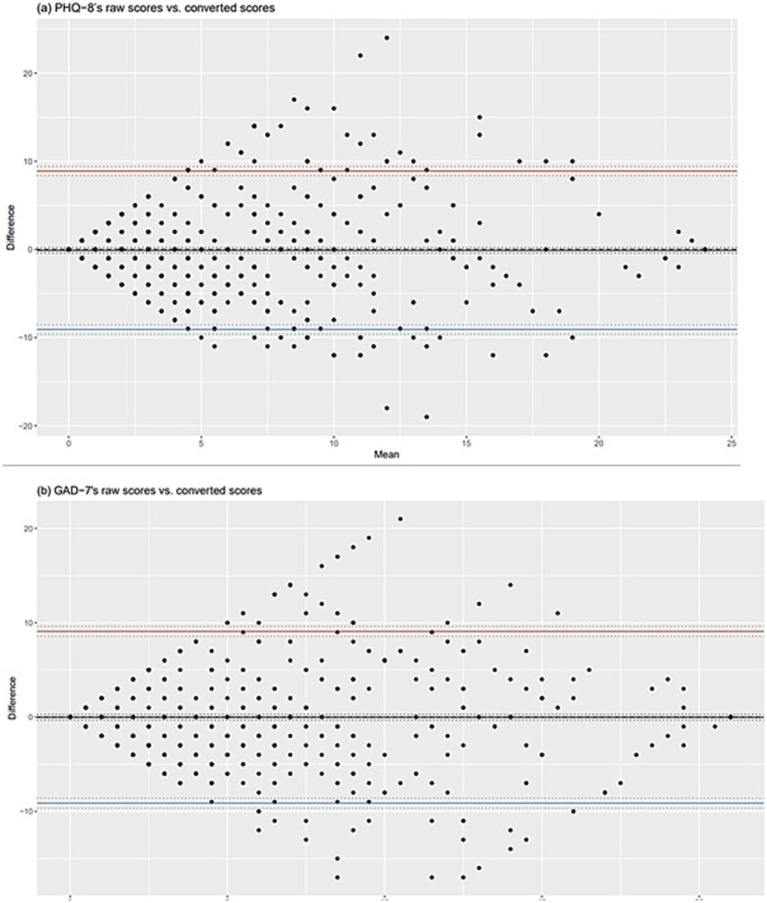
Bland–Altman plots between raw and converted scores of the PHQ-8 and GAD-7.

A crosswalk score conversion table of the DASS-21 depression to PHQ-8 and the DASS-21 anxiety to GAD-7 are provided in Table 4. The agreement between the PHQ-8’s raw and converted scores from DASS-21 depression and GAD-7’s raw and converted scores from DASS-21 anxiety showed good interrater reliability with ICC of 0.616 and 0.578, respectively. Figure 2 showed the Bland–Altman plots between the PHQ-8’s raw and converted scores from DASS-21 depression and GAD-7’s raw and converted scores from DASS-21 anxiety. The mean difference between the PHQ-8’s raw and converted scores from DASS-21 depression and between GAD-7’s raw and converted scores from DASS-21 anxiety was −0.09 and −0.02, respectively.

**Table 4:** Conversion table from the DASS-depression to PHQ-8 and from the DASS-anxiety to GAD-7.

Conversion for the PHQ-8		
Original DASS-depression score	Equivalent PHQ-8 score	Bootstrap SE
0	0	0.04
2	0	0.12
4	1	0.19
6	2	0.27
8	2	0.31
10	3	0.35
12	4	0.39
14	4	0.41
16	5	0.43
18	6	0.43
20	8	0.43
22	9	0.45
24	10	0.50
26	12	0.58
28	14	0.69
30	15	0.81
32	17	0.91
34	18	1.12
36	20	1.45
38	22	1.34
40	23	0.89
42	24	0.44

Conversion for the GAD-7		
Original DASS-anxiety score	Equivalent GAD-7 score	Bootstrap SE
0	0	0.03
2	0	0.07
4	1	0.13
6	1	0.16
8	2	0.20
10	3	0.26
12	4	0.32
14	5	0.38
16	6	0.45
18	7	0.52
20	8	0.58
22	10	0.63
24	11	0.68
26	13	0.73
28	14	0.77
30	16	0.81
32	17	0.82
34	18	0.80
36	19	0.72
38	20	0.59
40	21	0.40
42	21	0.15

## Discussion

The present study provides the psychometric performance of the DASS-21 in the youth population. Our results show that the DASS-21 is a useful tool to measure depression, anxiety, and stress in the youth population in Singapore. We found that its structural validity, measurement invariance across age groups and gender, reliability, and convergent validity with the WHODAS 2.0 were supported in this sample. In the confirmatory factor analysis, the goodness-of-fit results confirmed that the original three-factor model, as proposed by the developer, fits our data. Similar results were also found among male and female medical students in Iran, Chinese left-behind children in China and adolescents samples from high schools in four countries, including Australia, Chile, China and Malaysia (Lovibond and Lovibond, 1995; Chen et al., 2024; Jafari et al., 2017; Mellor et al., 2015; Wang et al., 2016). Further investigation on measurement invariance across age groups and gender using the MGCFA shows that the configural, metric, and scalar measurement invariance models strongly support the 3-factor structure model. Hence, we can conclude that the MGCFA supports the full measurement invariance of the DASS-21 across different age and gender in our sample. Our results are similar to Chen et al. (2024) study, which demonstrated strong measurement invariance of the DASS-21 across males and females. Another study also found strong measurement invariance of the DASS-21 among adolescents across Western and Eastern cultures (Mellor et al., 2015). These findings have practical implications for users who are interested in using the DASS-21, as the results seem to support the use of subscale scores for examining depression, anxiety and stress within subgroups and undertaking mean group comparisons across age and gender.

Cronbach’s alpha coefficient for the depression, anxiety and stress subscale was 0.91, 0.87, and 0.89, respectively. The value of the Cronbach’s alpha in our study was higher compared to those obtained by previous studies (depression: 0.72 to 0.88; anxiety: 0.75 to 0.81; stress: 0.70 to 0.88) (Anghel, 2020; Jovanović et al., 2021; Le et al., 2017; Mellor et al., 2015; Norton, 2007; Silva et al., 2016; Tully et al., 2009). However, the Cronbach’s alpha value for the depression subscale was slightly lower compared to those obtained by Evans et al. (2021) and Naumova (2022) (Cronbach’s alpha: 0.92 to 9.93). We assessed the convergent validity of the DASS-21 by examining the correlations between the DASS-21 subscales and the WHODAS 2.0. The results showed moderate correlations (depression, r = 0.46, anxiety, r = 0.46, and stress, r = 0.43). Similarly other studies that examined the convergent validity of the DASS-21 with other scales also found that the depression subscale moderately correlated with the Positive and Negative Schedule-Positive Affect and the Beck Depression Inventory (Norton, 2007; Jovanović et al., 2021); and anxiety subscale moderately correlated with the GAD-7 (Evans et al., 2021). To the best of our knowledge, this is the first study that provides a crosswalk conversion table of the DASS-21 depression to PHQ-8 and DASS-21 anxiety subscale to GAD-7 using the equipercentile method. The equipercentile linking method has been widely used to provide cross-walk scores for various measures in the field of psychiatry (Samara et al., 2014; Leucht et al., 2013; Leucht et al., 2012; Leucht et al., 2017; Furukawa et al., 2019; Levine et al., 2021; Abdin et al., 2024). Recently, the equipercentile linking method has been successfully used to provide a Clinical Interview Scheduled-Revised total score equivalent to the DASS-21 total score, in the Brazilian Longitudinal Study of Health (ELSA-Brasil) COVID-19 Mental Health Cohort study (Fatori et al., 2022). In our study, we found the ICC between the raw and converted PHQ-8 score was 0.616, while the ICC between the raw and converted GAD-7 score was 0.578, suggesting that the DASS-21 depression and the PHQ-8 as well as the DASS-21 anxiety and GAD-7 were practically exchangeable. We have also conducted linking analysis based on SEM and found the ICC was slightly lower than the equipercentile method (Supplementary Table 1). We suggest that the conversion tables can be used to measure prevalence and trends in depression and anxiety using both scales interchangeably in our youth sample. For example, using values from the conversion table of the DASS-21 depression to PHQ-8 to establish the prevalence of depression using a standard cutoff for PHQ-8 > 10, we will be able to show that the weighted prevalence of depression was available with marginal differences when derived either from PHQ raw or conversions score (11.6 versus 11 0.4%).

This study had some limitations. First, the data for this study were collected from a community-dwelling sample excluding those from hospitals and prisons, thus, the generalizability of our findings to other population including those from hospitals and prisons and patients with other mental disorders and chronic physical conditions needs further investigation. In the present study, although measures were administered in multiple languages (i.e., English, Mandarin, Bahasa Melayu and Tamil), the majority responded using the English version of the questionnaire. Hence, we specifically examined the psychometric properties of the English version of the DASS-21, and the validity and reliability of the instrument among those who were not fluent in English remains uncertain.

## Conclusion

The present study provides evidence of structural validity, full measurement invariance across age and gender, internal consistency, reliability, convergent validity and linking of the DASS-21 in the youth population. Hence, we suggest that the DASS-21 is a valid tool to measure depression, anxiety, and stress among youths in Singapore.

## Data Availability

Readers who wish to gain access to the data can write to the corresponding author with their requests. Access can be granted subject to the institutional review board (IRB) and the research collaborative agreement guidelines.
